# Optical–Structural Optimization for Condensation Suppression in Automotive Camera Modules

**DOI:** 10.3390/s25216515

**Published:** 2025-10-22

**Authors:** Kouwen Zhang, Yike Xu, Shenwei Xu, Xiaoyang Lin, Junyu Zhou, Zhaoqing Liu, Yan Li, Haoyun Wei

**Affiliations:** 1 State Key Laboratory of Precision Measurement Technology and Instruments, Department of Precision Instrument, Tsinghua University, Beijing 100084, China; zkw@sunnyoptical.com (K.Z.); lin-xy24@mails.tsinghua.edu.cn (X.L.); liyan@mail.tsinghua.edu.cn (Y.L.); 2 Zhejiang Sunny Smartlead Technology Co., Ltd., Ningbo 315400, China; wsykxu@sunnyoptical.com (Y.X.); swxu@sunnyoptical.com (S.X.); ryan.zhou@sunnyoptical.com (J.Z.); zlliuzqing@sunnyoptical.com (Z.L.)

**Keywords:** camera modules, condensation mitigation, automotive cameras, optical, structural optimization

## Abstract

Cameras have become indispensable sensors in intelligent vehicles, with their deployment steadily increasing across modern automobiles. It is critical for camera modules to have reliable and accurate environmental perception, but a major challenge is condensation inside the modules that severely compromises imaging quality. To address this issue, we performed comprehensive thermodynamics-based simulations to clarify condensation mechanisms and evaluate their impact on optical imaging performance. Based on these insights, we proposed an integrated optical–structural optimization strategy that reduces the internal cavity volume adjacent to the first lens, simultaneously increasing the first lens thickness and the curvature of its internal surface. This strategy both reduces water vapor volume and elevates the temperature of potential condensation zones. The optimized module exhibits markedly improved resistance to condensation compared with the baseline design in the experiment, raising the critical condensation threshold from a sudden temperature drop of 42 °C to over 60 °C. This approach effectively mitigates condensation under harsh environmental conditions without additional cost. Our simple yet effective design is broadly applicable to diverse automotive camera module architectures, thereby enhancing system reliability and improving the overall safety of autonomous driving.

## 1. Introduction

Perception is the core of autonomous vehicles (or Autonomous Driving Systems, ADSs) [1,2,3,4,5,6], which rely heavily on diverse sensors—including cameras [7], LiDAR [8,9], radar [10,11], and ultrasonic sensors [12], etc., to capture information about the surrounding environment [13]. When a vehicle is not equipped with perception modules such as cameras, drivers may have blind spots in their field of vision when turning, changing lanes, reversing, etc. The acquired environmental information not only serves as the basis for understanding driving scenarios but also facilitates the subsequent formation of driving decisions and execution of driving actions [14,15]. Among these sensors, automotive cameras are particularly critical due to their perception being similar to that of human eyes, making them highly compatible with traffic systems originally designed for human observation. Consequently, cameras have become indispensable sensing modules for autonomous driving [13]. There are differences in the configuration of automotive cameras under different levels of intelligent driving. At levels 1–2, a vehicle is generally equipped with front and rear cameras. At Level 3, a single vehicle is generally equipped with more than eight cameras for 360-degree blind-spot-free perception. Existing data shows that when a rear-view camera module is installed and functioning properly, it can reduce the reverse accident rate by 16%; when only a side-view wide-FOV module is installed in conjunction with a flat mirror, it can eliminate blind spots on both sides of a bus [16].

**Figure 1:**
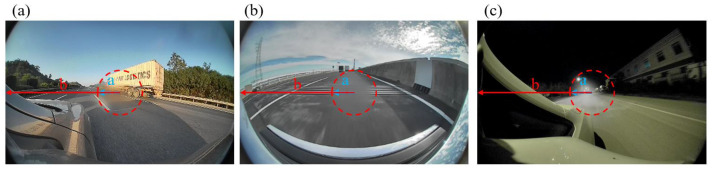
Impact of condensation on automotive camera imaging. (**a**) Condensation in high-speed scenarios; (**b**) condensation covering lane lines on urban roads; (**c**) condensation in nighttime conditions.

To enable long-range, high-definition perception and provide a larger time margin for decision-making, the resolution requirements of automotive cameras continue to rise, with eight-megapixel modules emerging as the mainstream. Meanwhile, cameras must remain reliable under industry-standard conditions that include wide temperature ranges, high heat and humidity, and rapid thermal fluctuations. A major obstacle is condensation within the module, which leads to hazy distortion in the central image region, as shown in Figure 1. Experiments demonstrate that this phenomenon severely impairs optical performance, obscuring critical details such as road markings and traffic signs—particularly in high-resolution modules. Such degradation directly hinders autonomous driving decisions and compromises safety, making condensation one of the most pressing challenges in the application of automotive cameras.

An automotive camera module (ACM) typically comprises a sensor chip, lens assembly, supporting electronic components, and an outer housing. To meet the IP69 protection standard, a sealing ring is incorporated around the lens to block external moisture ingress [17]. Although this design prevents external moisture from entering the camera, the complex internal structure inevitably creates irregular yet interconnected cavities that allow internal moisture migration and accumulation. Under rapid environmental changes, these cavities may locally reach saturation, leading to condensation formation. Several anti-condensation strategies have been investigated. In surface treatment technology, there are studies on anti-fog coatings and nanometer coatings. However, these techniques are not verified in high optical performance and strict operating temperature requirements of camera modules, and they lead to higher costs [18,19,20]. Other methods include injecting dry gas in front of the lens to limit moisture contact, preheating the lens, or performing cleaning operations prior to use [21,22,23]. Currently, the dominant approach relies on heating to reduce the temperature gradient across the lens. One method utilizes the ACM’s inherent rapid heating and high-temperature characteristics by designing a thermal conduction structure that transfers heat from the module to the outermost lens. The heat then propagates outward through the glass, raising the external surface temperature to suppress condensation [24,25]. However, this method is ineffective when the module is just powered on, or under sudden temperature drops. Another approach integrates temperature and humidity sensors with a control unit inside the ACM. By continuously monitoring environmental conditions, the system determines when to activate the heating module for lens warming [26,27]. Although effective, this method introduces additional sensors and control circuits, significantly increasing cost and enlarging the module, which runs counter to the trend toward miniaturization. To date, however, little research has explored the method of exploiting the optomechanical characteristics of the optical system itself as a means to mitigate condensation.

In this study, we propose an optical–structural optimization strategy that effectively suppresses condensation in ACMs without increasing system complexity or cost. This method jointly optimizes the ACM’s optical and structural parameters based on thermodynamic simulations of condensation and its impact on imaging quality. We reduced the volume of the internal cavity of the first lens and its connected cavities and simultaneously increased the first lens thickness and the curvature of its internal surface so that condensation is significantly mitigated. Using this strategy, a high-resolution automotive camera was designed and manufactured. Experimental results show that, compared with the baseline design, the optimized module raises the critical condensation temperature difference from 42 °C to over 60 °C while maintaining a modulation transfer function (MTF) that meets operational requirements, i.e., above 0.5 @ 83 lp/mm. This simple yet effective solution addresses industry demands for low-cost, compact designs while substantially enhancing performance, aligning with the evolving needs of key sensing components in autonomous vehicles.

## 2. Simulation of Condensation in ACM

### 2.1. Materials and Methods

**Figure 2:**
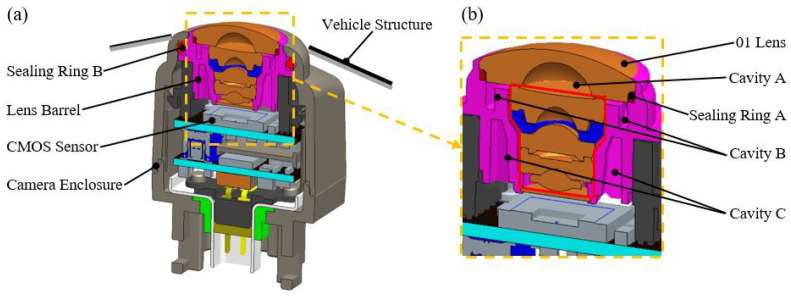
(**a**) Cross-sectional view of a typical ACM; (**b**) view of the optical section.

To investigate condensation in ACMs, simulations were conducted using a module developed by our team that meets Tier-1 customer requirements, as shown in Figure 2. This ACM is a 360° surround-view module. The lenses are produced by Ningbo Sunny Automotive Optics Technology Co., Ltd. (Ningbo, China), and the chip used is produced by Sony Corporation of Japan (Tokyo, Japan). The photographing distance of this ACM is 120 mm∼*∞*, and this ACM is designed, produced, and manufactured by Zhejiang Sunny Smartlead Technology Co., Ltd. (Yuyao, China) The module comprises the imaging lens group and its barrel structure, the CMOS sensor, and the camera housing. Specific information about the materials is provided in Table 1. Two sealing rings are included: sealing ring A closes the gap between the external optical component (the 01 lens) and the lens barrel, while sealing ring B seals the gap between the barrel and the housing.

This design achieves IP69 protection and effectively prevents moisture exchange between the interior and exterior. As shown in Figure 2b, the barrel contains cavity structures such as Cavity A, Cavity B, and Cavity C, which are generated during the molding process solely to satisfy structural strength and material-saving requirements. The entire ACM is assumed to be assembled under controlled conditions of a temperature in the range of 20–25 °C and relative humidity (RH) in the range of 40–60%, which represent typical environmental parameters on the production line. The main process flow of this ACM includes cleaning, active alignment, baking, semi-finished product testing, upper shell component assembly, upper and lower shell assembly, ultrasonic welding, airtightness testing, bracket assembly, finished product testing, etc. All the key equipment used is designed and developed by Sunny Group.

To evaluate the influence of condensation on imaging, an analysis of the generation principle of condensation was conducted regarding temperature and humidity. The temperature and moisture flow in the ACM were simulated using FLOEFD software (Version 2022.1, Siemens Digital Industries Software, Plano, TX, USA). During simulation modeling, we fully considered the impact of heat conduction and the energy changes during the process of internal water vapor changing from a gaseous to liquid state when condensation occurs. According to the analysis of the first law of thermodynamics, there are mainly two heat transfer modes in the lens: heat conduction in solids and heat convection in cavity gases. At room temperature, when the module is powered on, internal electronics generate heat and raise the sensor temperature to approximately 50 °C. So, the simulation started with an initial internal temperature of 50 °C, an internal relative humidity of 11% (corresponding to 60% RH at 20 °C), and a chip power of 0.4 W.

**Figure 3:**
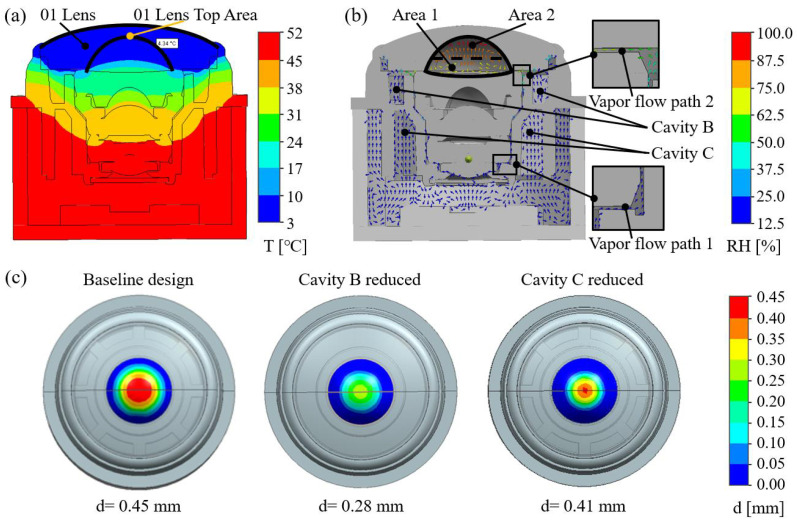
Simulation results of the baseline camera module. T: temperature; RH: relative humidity; d: condensation thickness. (**a**) Thermal distribution; (**b**) water vapor distribution and transport; (**c**) condensation on internal surface of 01 lens after 40 s. From left to right: the baseline design, the baseline design with cavity B removed, and the design with cavity C removed.

In real vehicle applications, only the area surrounding the 01 lens is exposed, while the remainder of the module is enclosed by vehicle structures. To replicate a worst-case scenario—such as a vehicle transitioning from a warm indoor setting to a cold outdoor environment at 0 °C—the exterior boundary was set to 0 °C, causing the 01 lens surface temperature to instantaneously drop to 0 °C. The subsequent temperature distribution and vapor behavior were then examined. During the simulation, the mesh and time step were carefully evaluated. The mesh was set to be divided with 0.1 mm for the solid region and 0.05 mm for the fluid region, with a total of 4.49 million mesh elements and a time step of 1 s. The simulation results are presented in Figure 3. As shown in Figure 3a, the temperature distribution exhibits a pronounced gradient from the top to the bottom of the module. The coldest region is at the inner apex of the 01 lens, with a temperature of only 4.3 °C, while the hottest region is at the bottom of the module near the CMOS sensor, exceeding 45 °C. Notably, in the cavity A area, the gradient is particularly steep, with a temperature difference of more than 20 °C between the inner apex of the 01 lens and the front surface of the 02 lens. Figure 3b presents the water vapor flow field and its relative humidity distribution. In cavity A, the relative humidity of the enclosed air rises rapidly due to the low local temperature. Meanwhile, the strong temperature gradient within the module drives water vapor from cavities B and C through the gap between the 01 and 02 lenses into this cavity A, further elevating the relative humidity and ultimately leading to condensation. Simulation results show that in the upper half of cavity A (above the dashed line), water vapor reaches saturation, causing condensation near the apex of the 01 lens’s inner surface. As condensation occurs, the relative humidity in this region decreases.

The dynamic condensation process can be clearly illustrated. Here, the left image in Figure 3c shows the condensation distribution on the 01 lens after 40 s. Condensation covers roughly one-third of the lens diameter, with the maximum film thickness of 0.45 mm at the inner apex. Through the above simulation combined with the principle analysis of dew formation, reducing the humidity of the internal cavity can alleviate condensation. The humidity of the internal cavity is related to the size of the internal cavity volume. However, since there are many internal cavity volumes inside the camera module, further research on the significant areas is needed. To further investigate the contribution of vapor transport, additional simulations were conducted with cavities B and C removed individually. Results show that eliminating cavity B significantly reduces the film thickness to 0.28 mm, whereas the reduction in cavity C results in a less pronounced reduction to 0.41 mm. This is attributed to the higher overall temperature of cavity A and its longer vapor transport path to cavity A (vapor flow path 1 in the figure), which provides partial isolation of moisture transfer. Through the above analysis, the structural optimization strategy of removing cavity B has the most significant impact on reducing internal humidity.

### 2.2. Simulation Analysis of Condensation-Induced Optical Imaging Degradation

**Figure 4:**
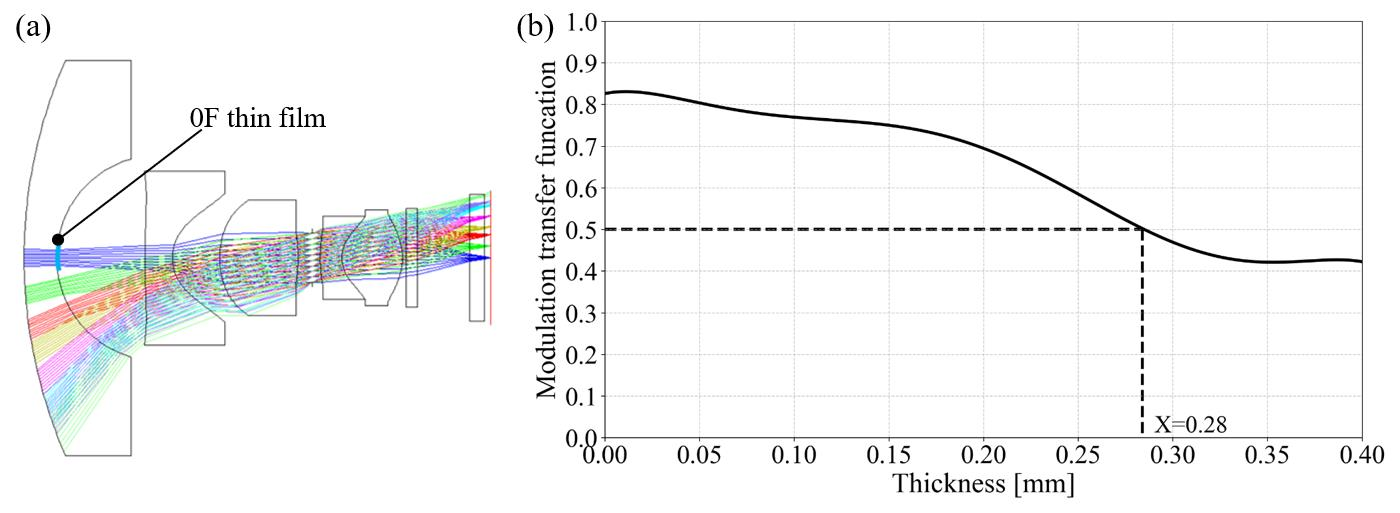
Optical simulation for condensation effects. (**a**) Optical simulation design; (**b**) MTF results for 83 lp/mm with different condensation thicknesses.

It is evident that condensation leads to a decline in ACM imaging quality. To quantify this effect, we further performed optical imaging analysis using Zemax (Version 19.4, Ansys, Canonsburg, PA, USA) and, based on industry specifications for surround-view module resolution, adopted the criterion of MTF > 0.5 at half the Nyquist frequency (83 lp/mm) as the threshold for acceptable imaging quality. Figure 4a shows the lens model in Zemax, where the aperture stop is located between the third and fourth lenses. Combining the previously analyzed condensation distribution with Zemax ray-tracing simulations, it can be found that condensation primarily affects the central 0° field of view (FOV), with the impacted range covering about 15% of the full FOV. Since the apex of the 01 lens has the thickest condensation layer, it poses the greatest risk to image quality. In the simulation, varying water film thicknesses were applied at the zero field of view from 0 mm to 0.40 mm to represent different levels of condensation. The results in Figure 4b show that when the condensation layer is thinner than 0.20 mm, the MTF for a spatial frequency of 83 lp/mm decreases gradually. However, once the thickness exceeds 0.20 mm, the MTF drops sharply, reaching ∼0.42 at around 0.32 mm and then stabilizing. The MTF of 83 lp/mm falls to 0.5 when the condensation thickness reaches 0.28 mm, which no longer meets the imaging quality requirement. Therefore, in design optimization, the condensation layer thickness should be limited to less than 0.28 mm.

## 3. Optical–Structural Optimization Design and Simulation Verification

### 3.1. Optical–Structural Optimization Design

Based on the condensation characteristics analysis, we proposed a simple but effective method to mitigate condensation effects using the optical–structural optimization strategy without adding extra components. First, the simulation results (Figure 3b) indicate that water vapor from all internal cavities can be transported into cavity A, making the reduction in overall vapor volume the key to humidity control. Since additional dehumidification measures are impractical in the assembly environment, the feasible approach is to minimize cavity volume. As shown in Figure 3c, although cavity C has a relatively large volume, its long transport path and weak convection make its impact on cavity A limited; in contrast, cavity B contributes significantly to cavity A and is therefore removed in the optimized design. Second, raising the inner surface temperature of the 01 lens and reducing its thermal gradient can lower the probability of condensation and disperse the condensation-affected region, thereby significantly reducing film thickness when condensation occurs. To achieve this while maintaining the module’s external dimensions, the central thickness of the 01 lens is increased and its inner surface curvature radius is enlarged, which also further reduces the volume of cavity A.

**Figure 5:**
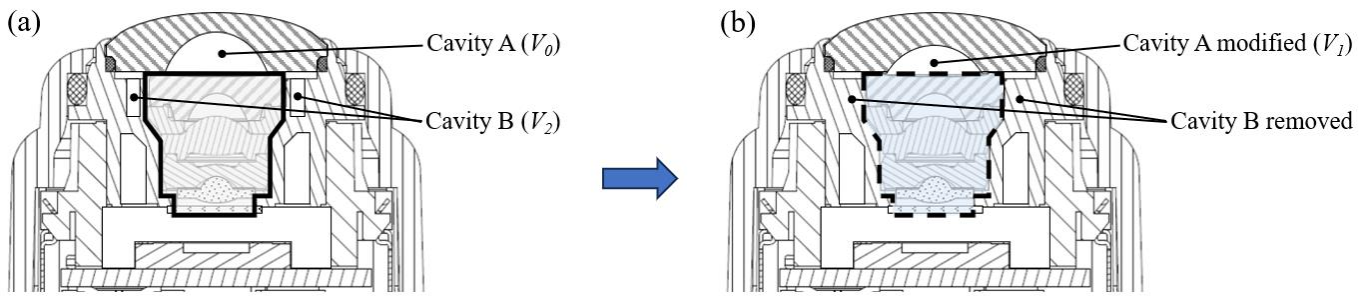
Camera module structure and optimization design. (**a**) Module structure before optimization design, which serves as a baseline design for later simulation and experiments. (**b**) Module structure after optimization. Cavity B connected to the 01 lens is canceled, and cavity A under the 01 lens is reduced from *V*_0_ to *V*_1_.

Following this approach, Figure 5 illustrates the proposed optical–structural optimization design: the 01 lens central thickness is increased from 0.9 mm to 1.6 mm, its inner surface curvature radius is increased from 2.85 mm to 3 mm, and the volume of cavity A is reduced from 36.7 mm^3^ (*V*_0_) to 28.4 mm^3^ (*V*_1_). At the same time, cavity B—directly connected to the 01 lens in the baseline design—is filled, eliminating approximately 64.3 mm^3^ (*V*_2_) of air volume. The remaining parts of the lens group are only slightly adjusted to ensure that overall imaging quality remains essentially unchanged under normal operating conditions.

### 3.2. Comparison Analysis of ACM Before and After Optimization

**Figure 6:**
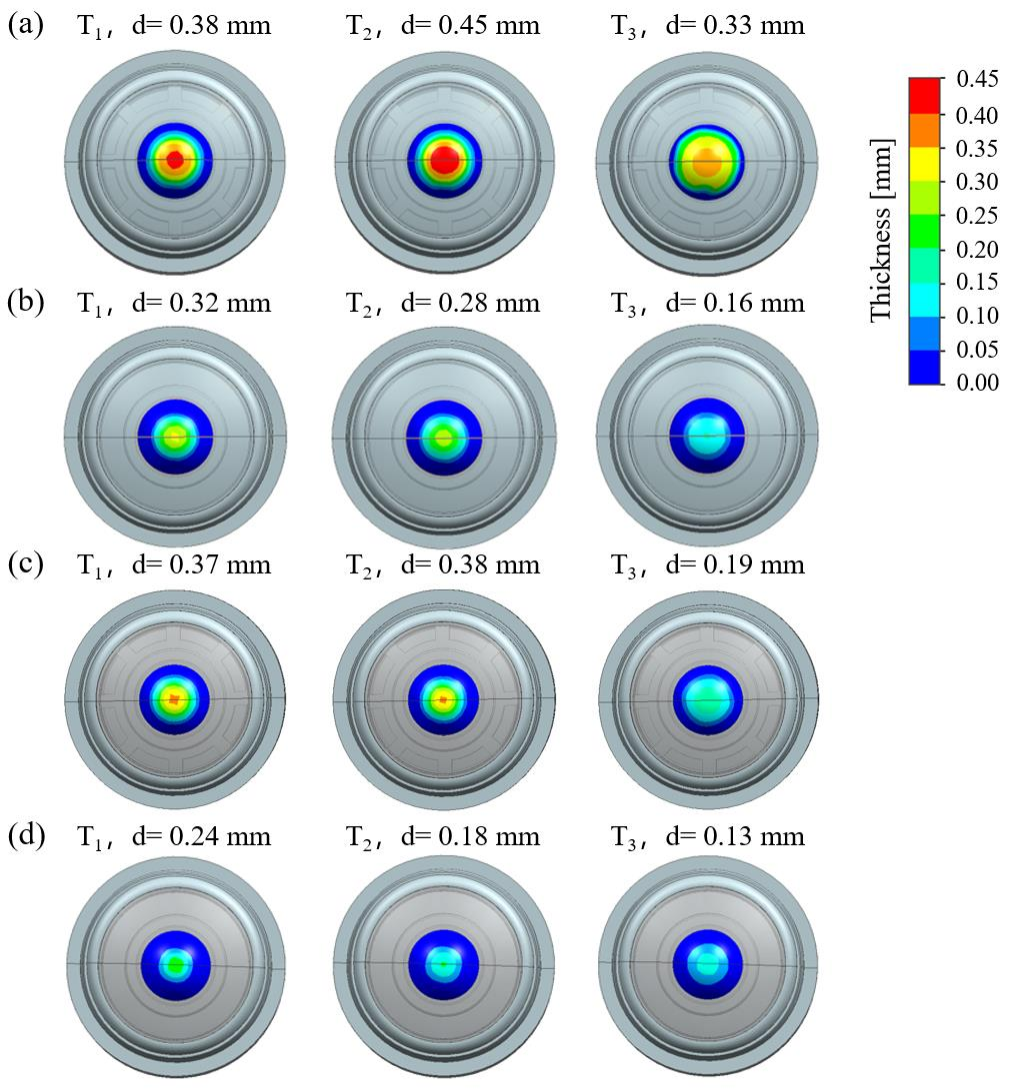
Simulation results of condensation thickness on the inner surface of the 01 lens at specific times. Time after instant cooling is represented as T_1_: 20 s, T_2_: 40 s, and T_3_: 120 s. (**a**) BD case. (**b**) MD1 case. (**c**) MD2 case. (**d**) FOD case.

To verify the effectiveness of the proposed optimization strategy, comparative simulations were performed for the baseline design (BD), the modified design with cavity B removed (MD1), the modified design with a reduced cavity A volume (MD2), and the fully optimized design (FOD). The simulation inputs were kept consistent with those used in the previous analysis. Each simulation covered a dynamic period of approximately 120 s, spanning the transient environmental change through the steady-state system response. Representative condensation states at 20 s, 40 s, and 120 s are shown in Figure 6, where rows (a)–(d) correspond to the BD, MD1, MD2, and FOD cases, respectively.

Comparing MD1 and MD2 with BD, both modifications reduce condensation thickness and affected area, confirming that each measure has a positive effect in alleviating condensation. However, it is also evident that neither measure alone is sufficient to maintain the condensation thickness below the critical threshold for acceptable imaging performance. At 20 s and 40 s, the maximum film thicknesses of both MD1 and MD2 remain above 0.28 mm. By contrast, the FOD result exhibits markedly superior condensation suppression, with significantly reduced thickness and affected area across all stages. For example, at 20 s, the maximum condensation thickness is only 0.24 mm, representing reductions of 37%, 25%, and 35% relative to BD, MD1, and MD2, respectively. The condensation-affected area (thickness > 0.05 mm) is about 2.25 mm^2^, corresponding to reductions of 66.7%, 49.0%, and 53.5% relative to the three other designs.

**Figure 7:**
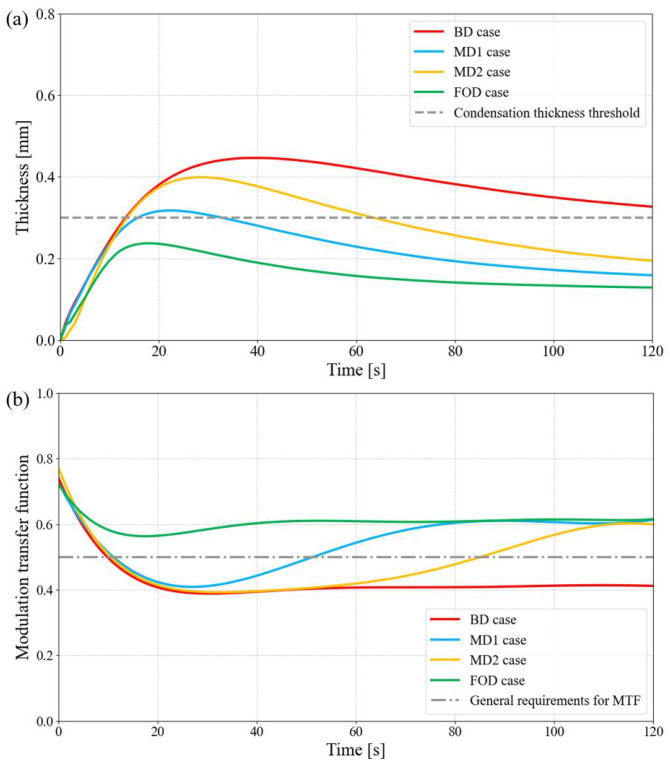
Simulation evolution curve of condensation thickness and imaging MTF. (**a**) Condensation thickness evolution of 01 lens inner apex; (**b**) 83 lp/mm transfer function evolution of 0° FOV affected by condensation thickness evolution in (**a**).

To further consider the full temporal evolution, Figure 7a presents the condensation thickness at the inner apex of the 01 lens over 120 s. The maximum thicknesses for BD, MD1, MD2, and FOD are 0.44 mm, 0.32 mm, 0.40 mm, and 0.24 mm, respectively. Moreover, the durations during which the condensation exceeded the 0.28 mm threshold are >110 s, ∼20 s, ∼50 s, and 0 s, respectively, demonstrating the FOD design’s effectiveness in avoiding performance-limiting condensation. Using the thickness data from Figure 7a, the MTF at 83 lp/mm for a 0° FOV was further simulated, as shown in Figure 7b. The FOD design consistently maintains an MTF above 0.55 throughout the analysis period, indicating that condensation has a negligible impact on imaging quality. In contrast, the BD, MD1, and MD2 cases fall below the 0.5 threshold for >120 s, ∼40 s, and ∼80 s, respectively, each experiencing significant image degradation due to condensation. These results clearly show that the proposed simple yet effective optimization strategy can robustly prevent condensation from impairing imaging performance.

## 4. Experiment Results

Based on the above simulation analysis, we fabricated ACM prototypes designed according to the FOD scheme using the same assembly environment as the BD reference modules. To verify the practical effectiveness of the optimized design, both sets of modules were first pre-heated in a thermal oven. The temperature control accuracy of the oven used is within ±1 °C. Once the modules reached the target temperature, they were removed and immediately immersed in a 0 °C ice–water mixture to replicate the low-temperature exposure encountered when a vehicle transitions abruptly from a warm indoor environment to cold outdoor conditions. The oven temperature was varied from 25 °C to 70 °C to simulate transient cooling processes under different initial states. Following the results in Figure 7a, to capture the worst condensation conditions of the FOD ACM, immersion time in the ice–water mixture was fixed at 15 s. After removal, the modules were rapidly transferred either to a microscope for condensation morphology observation or to a test setup for standard image capture to evaluate imaging performance. The final transfer function results are obtained through the commonly used Imatest (Version 24.1.3 x64, Imatest LLC, Boulder, CO, USA ) software in the industry.

**Figure 8:**
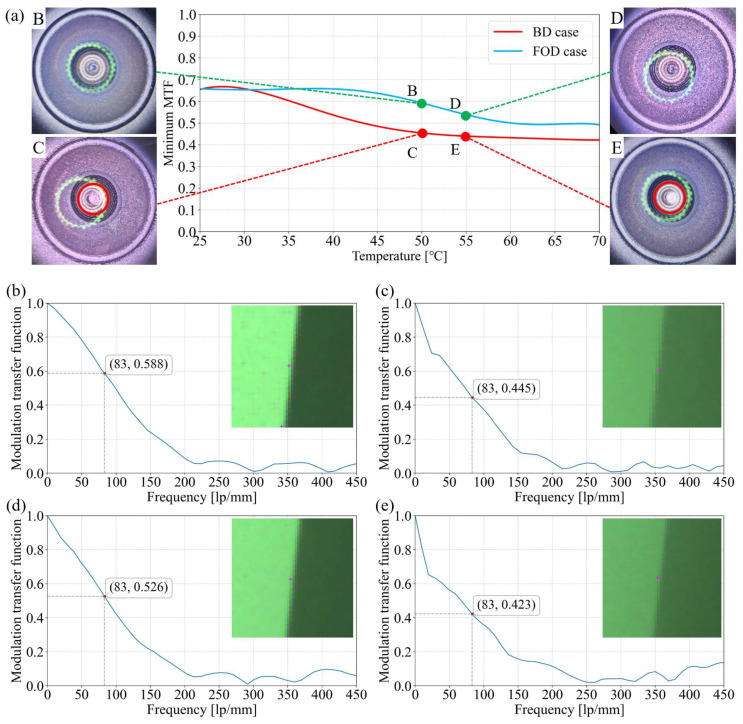
Experimental results. (**a**) Fitted MTF curve for different temperatures of BD and FOD ACMs. B, D, C, and E are four typical points, each with a micrograph attached. (**b**–**e**) MTF of points B, C, D, and E, respectively. Typical MTF values at 83 lp/mm are marked in each plot.

The experimental results are summarized in Figure 8. The central plot of Figure 8a shows the minimum MTF values measured at 83 lp/mm. A clear divergence between the BD and FOD ACM is observed once the temperature difference exceeds 30 °C. For the BD ACM, condensation onset occurs around ΔT = 42 °C, where the MTF drops below the 0.5 threshold, leading to significant degradation in imaging quality. In contrast, the FOD module maintains a relatively stable MTF up to ΔT ≈ 45 °C. Only beyond this point does the MTF begin to decline due to condensation, reaching ∼0.5 at ΔT ≈ 60 °C and remaining near this level even up to ΔT = 70 °C. Microscopic observations further confirm these findings. As shown in the side images of Figure 8a, at ΔT = 50 °C and 55 °C, the BD ACM lens exhibits obvious whitening due to condensation, as seen in micrographs C and E. By contrast, the FOD ACM lens shows no visible condensation features under the same conditions, as illustrated in images B and D. Here, the green circular fringes visible in the micrographs correspond to illumination artifacts from the microscope. Consistent with these observations, Figure 8b–d present a comparison of the corresponding MTF curves and captured images, further demonstrating the superior imaging stability achieved by the FOD ACM.

These results demonstrate that the critical temperature differences for both condensation onset and unacceptable imaging degradation are increased by approximately 50% in the FOD design. Notably, the FOD module can sustain imaging performance near the 0.5 threshold over a ΔT range of nearly 70 °C, effectively covering most practical condensation scenarios and substantially enhancing ACM robustness.

## 5. Conclusions

This study addresses condensation in ACM under real operating conditions. Unlike conventional anti-condensation methods that rely on heating, our approach leverages optical–structural optimization to mitigate condensation formation. Based on thermodynamic and condensation principles, multi-dimensional simulations of temperature, vapor transport, and MTF were carried out. Taking cost, manufacturability, and module size into account, we developed an optical–structural optimization strategy that effectively suppresses condensation. The optimized design reduces the cavity volume beneath the 01 lens by increasing both its central thickness (from 0.9 mm to 1.6 mm) and inner surface curvature radius (from 2.85 mm to 3 mm) while removing the nearby cavity in the house module, resulting in a total volume reduction of 72.6 mm^3^. Experimental validation confirmed that the strategy shortens the time needed to reach the most severe condensation condition, and the critical temperature differences for both condensation onset and unacceptable imaging degradation are increased by approximately 50% in the FOD ACM, ensuring stable imaging quality under most extreme conditions. The proposed design has passed the rigorous reliability tests for automotive-grade products, indicating that it has a stable stress structure.

This simple solution achieves significant technical gains without increasing cost or altering module dimensions. It offers strong universality, making it adaptable to diverse camera module types, and provides a scalable framework for future high-resolution designs. By effectively mitigating condensation, the proposed design enhances imaging stability and reliability, addressing the stringent safety requirements of intelligent and autonomous driving systems. In the future, we will conduct refined stress analysis and research focused on further expanding the non-condensation temperature range to meet the usage requirements of special extreme scenarios, in line with the industry trends of overall automotive price decline and low-cost camera modules.

## Figures and Tables

**Table 1:** Materials and characteristics of main components of ACM.

Component	Material	Thermal Condition (W/m·K)	Density (g/cm^3^)	Specific Heat Conductivity (KJ/Kg·K)
Lens barrel	Plastic	0.13	1.65	1.15
01 Lens	Glass	0.91	4.24	0.5
Other lens	Plastic	0.17	1.08	2.5
Upper Shell	Plastic	0.13	1.56	1.1
Lower Shell	Plastic	0.13	1.56	1.1
O-ring	Rubber	0.25	1.13	0.15

## Data Availability

The original contributions presented in this study are included in the article. Further inquiries can be directed to the corresponding author.

## Abbreviations

The following abbreviations are used in this manuscript:

ADS	Autonomous Driving Systems
ACM	Automotive Camera Module
MTF	Modulation Transfer Function
RH	Relative Humidity
FOV	Field of View
BD	Baseline Design
MD1	Modified Design 1
MD2	Modified Design 2
FOD	Fully Optimized Design
